# The Australia and New Zealand clinical quality registry for the treatment of eating disorders (TrEAT Registry): protocol and preliminary data

**DOI:** 10.1186/s40337-025-01506-5

**Published:** 2025-12-24

**Authors:** Deborah Mitchison, Christopher Basten, Kyra Bennett, Megan Bray, Sue Byrne, Mandy Goldstein, Phillipa J. Hay, Gabriella Heruc, Katie McGill, Katarina Prnjak, Marion E. Roberts, Kris Rogers, Patrick Russell, Niamh Taggart, Jack Tame

**Affiliations:** 1 Body image and Eating Network (BEAN), Graduate School of Health, Faculty of Health, University of Technology Sydney, Sydney, Australia; 2 Basten & Associates Clinical Psychologists, Westmead, Australia; 3 Eating disorders and Nutrition Research Group (ENRG), Eating Disorders and Body Image (EDBI), Translational Health Research Institute, School of Medicine, Western Sydney University, Penrith, Australia; 4 Collective Health Co, Sunshine Coast, Australia; 5 Western Australia Department of Health, Perth, Australia; 6 Richardson Group, West Perth, Australia; 7 everyBody Psychology & Wellbeing, Bondi Junction, Australia; 8 Eating Disorders and Body Image (EDBI), Translational Health Research Institute, Western Sydney University, Penrith, Australia; 9 ReCentre, Waratah Private Hospital, Hurstville, Australia; 10 Appetite for Change, Sydney, Australia; 11 Hunter New England Local Health District, Waratah, Australia; 12 University of Newcastle, Callaghan, Australia; 13 School of Population Health, Faculty of Medical & Health Sciences, University of Auckland, Auckland, New Zealand; 14 Nurture Psychology, Auckland, New Zealand; 15 Department of Internal Medicine, Royal Adelaide Hospital, Port Road, Adelaide, South Australia Australia

**Keywords:** Eating disorders, Clinical quality registries, Health surveillance, Quality improvement, Australia, New Zealand

## Abstract

**Background:**

Eating disorders are a major public health concern in Australia and Aotearoa New Zealand, with significant morbidity, mortality, and economic burden. Despite substantial government investment in eating disorder care, there is limited infrastructure to evaluate treatment outcomes, particularly in community settings. Clinical Quality Registries (CQRs) offer a mechanism for systematic data collection, benchmarking, and feedback to improve care quality. The Australia and New Zealand Clinical Quality Registry for the Treatment of Eating Disorders (TrEAT Registry) was developed to address this gap.

**Methods:**

The TrEAT Registry is a multi-centre, longitudinal CQR that collects clinician- and client-reported data across outpatient, day patient, residential, and inpatient settings. Data are collected at treatment commencement, during treatment, and at discharge or follow-up. Clients aged 13 years and older provide informed consent to contribute de-identified data to a research databank. Core measures include the Eating Disorder Examination Questionnaire (EDE-Q), Clinical Impairment Assessment (CIA), and Depression Anxiety and Stress Scale (DASS-21).

**Results:**

Between September 2021 and June 2025, 754 clients were invited to contribute their clinical data to the registry databank, with 88.1% consenting and 93.7% of consenting participants completing the pre-treatment survey. The sample was predominantly female (91.8%), young (mean age = 26.0 years), and urban-dwelling (> 85%). Most clients in the registry were treated in privately operated outpatient settings. Mean scores on the EDE-Q (Global = 3.80), CIA (Total = 30.92), and DASS-21 subscales (Depression = 10.29, Anxiety = 7.00, and Stress = 10.67) indicated clinically significant symptomatology.

**Conclusions:**

The TrEAT Registry is a pioneering initiative in eating disorder care, providing infrastructure for health surveillance, quality improvement, and research. The registry has supported real-world research and clinical trials, including ongoing evaluations of residential and virtual day programs, and planned evaluation of Medicare and credentialing systems. Its unique inclusion of private sector clinics and client consent enhances ethical standards and data richness. Planned expansion and digital enhancements aim to improve coverage, data accessibility, and follow-up rates, supporting a learning health system across Australia and New Zealand.

*Trial registration* Registered on the Australian Register of Clinical Registries (#ACSQHC-ARCR-279).

## Abstract (plain language summary)

This paper describes the TrEAT Registry, an initiative in Australia and New Zealand that collects information about people receiving treatment for eating disorders. The goal is to improve care by understanding what treatments work best, for whom, and in which settings. The registry includes people from both public and private clinics, and covers different types of care, such as outpatient, day programs, and residential services.

People who join the registry give permission for their treatment information to be used for research and quality improvement. This helps clinicians and researchers learn more about eating disorders and how to support recovery. The registry also gives feedback to clinics to help them improve their services.

So far, hundreds of people have joined the registry, most of whom are young and receiving care in outpatient settings. The registry is helping to identify gaps in access to care and will support future research and policy decisions. Over the next few years, the registry will expand to include more clinics, including more coverage of inpatient care, and improve how data is collected and shared.

## Background

*“Without data*,* you’re just another person with an opinion”* (words attributed to W. Edwards Deming, father of the quality movement, 1900–1993).

### The value of clinical quality registries in eating disorders

Clinical quality registries (CQRs) are the backbone of quality improvement in healthcare systems. CQRs collate data on client outcomes across services and provide benchmarked summaries of these data to clinicians and other end-users (e.g., government agencies). By doing so, they are a central mechanism in a “learning health care system” [32], supporting the monitoring of treatment safety and effectiveness to optimise outcomes and reduce costs. When these data are shared with clinicians and clients in a timely and interpretable format, CQRs also support shared clinical decision-making on an individual basis. Furthermore, CQRs are an important tool for real-world research, by providing central infrastructure to registry-based clinical trials [3, 37] and other research. The Australian Commission on Safety & Quality in Health Care (ACSQHC) highlights the essential role of CQRs, stating that they are:

*“…an important part of healthcare safety and quality improvement; a patient and consumer right; an essential professional requirement and health data custodian obligation.”* [6].

In mental health, coordinated and digitised clinical data collection, collation, and analysis is yet to become widespread standard practice, and consequently there is a relative dearth of mental health-related CQRs. In Australia, there are just five mental health registries listed on the national register of clinical registries, and aside from the registry that is the subject of this protocol, none that cover community treatment. Yet monitoring the nature of, and outcomes for, community treatment is essential to support mental health surveillance, as this is where most mental health care occurs. Privately operated treatment is particularly important in Australia given most community based mental health care is facilitated through the Government-funded Medicare system which subsidises > 12 million mental health services per year [5]. This is also true in Aotearoa New Zealand where, despite no government funding for private care, the vast majority of the mental health workforce (e.g., 85% of registered clinical psychologists) also operate in the private sector (NZPB, [30, 38]). Despite this clear utility of the private sector, private treatment sites are less frequently inluded in registry-based activities, due in part to the challenges with coordinating across sites with vastly different organisational structures [c^2^].

To encourage the development of registries, the Australian government has developed a National CQR Strategy [7] and Framework [1], and made mental health a Priority Domain [2]. Given their substantial and far-reaching impact, eating disorders are an area of mental health which could greatly benefit from a CQR. Yet, with the exception of registries in Nordic countries (e.g., Riksät in Sweden, which has been running for 30 years and 90% coverage of eating disorder treatment services [10]), eating disorder registries in most parts of the world are rare. Under the National Strategy, CQRs are prioritised for conditions that have the greatest burden, cost and unwarranted variance in outcomes [7]. The most recent estimates in Australia show that in 2023 alone, 1.1 million Australians had an eating disorder, resulting in 1,273 deaths, and costing the nation $66.9 billion [14]. Globally, eating disorders are documented to be a major burden of disease, accounting for 55.5 million cases worldwide in 2019 and associated with 6.6 million disability-adjusted life years [36]. There is also room for improvement in eating disorder care, with meta-analyses documenting remission rates for the best available community treatments to sit between 28 and 49% under ideal randomised controlled trial (RCT) conditions [22, 28].

Another reason to prioritise a CQR for eating disorders is that there is significant government investment in eating disorder healthcare in Australia that is currently under-evaluated. For example, in 2019 eating disorder specific Medicare items were implemented supporting those experiencing eating disorders to access quadruple the number of subsidised sessions per year (up to 40 mental health and 20 dietetic) that can usually be accessed for mental health care. While an evaluation of the uptake and reach of the scheme has been conducted, an in-depth clinical evaluation as part of this report was thwarted by a lack of symptom level data [24]. From 2021 to 2024, the Australian Government also funded the development and implementation of a credentialing system for clinicians trained in eating disorder management; but without a planned evaluation of its effectiveness in improving treatment outcomes [19]. Furthermore, new publicly funded residential eating disorder services are being rolled out in every State across Australia but without a coordinated evaluation plan [11]. A CQR that can collate high-quality, real-world clinical data across eating disorder clinics would help clinicians and policy workers to identify which treatments work best, under what settings, and for whom, ultimately supporting a self-improving and cost-efficient system of care. Thus, the value of an eating disorders CQR for Australia and New Zealand is evident.

### The development and growth of the TrEAT registry

In 2016, seeing a need and an opportunity to collate clinical outcomes data across eating disorder clinics, a group of eating disorder clinicians and clinician-researchers working in private practice in Sydney, Australia, collaborated to establish a minimum dataset (MDS) and digital data collection system (with digital feedback to clinicians), to support outpatient treatment of people with eating disorders. Ten years later, the Australia and New Zealand CQR for Eating Disorders (hereto referred to as the “TrEAT Registry”) was established as a CQR guided by standards in the National Framework [1] and has expanded to include a broad range of treatment settings (outpatient, day patient, residential and inpatient treatment). Treatment settings are both privately and publicly operated, and situated across four Australian states (New South Wales, Western Australia, Queensland, Victoria) and the North and South Islands of New Zealand (Auckland, Tauranga, Wellington, Christchurch). Over the past 10 years, the registry has supported thousands of clients. In 2024 alone, 267 new clients entered the registry with diverse eating disorder presentations (33% anorexia nervosa, 13% bulimia nervosa, 17% binge eating disorder, 37% other eating disorders), treated by 65 clinicians. Most clients in the registry are young, with 63% < 25 years. Studies using TrEAT Registry data, co-authored with registry clinicians, show that clients in the registry experience an eating disorder for an average of 2–10 years before accessing treatment [17] and have significant psychiatric comorbidity [12]. The registry has also provided infrastructure for major clinical trials, including the evaluation of Australia’s first residential eating disorder service, Wandi Nerida [13]. The TrEAT Registry is the only registry currently covering community-based eating disorders treatment in the Australasian region, and complements the work of the Lily Registry, which covers treatment of medically unstable inpatients who are admitted to hospital with an eating disorder [34].

### Aims

With the support of funding from the Australian Government, plans for the TrEAT Registry over the next five years to 2030 include significant national expansion to increase coverage across Australia and New Zealand. This paper details the protocol for the TrEAT Registry, providing end-users (people with eating disorders and their carers, clinicians, service managers, policy workers and researchers) with a record of the registry’s purpose and methods. We also provide a descriptive snapshot of clients who have enrolled in the research databank of the registry.

## Methods

### Design

The TrEAT Registry is a CQR, meaning that it not only collates clinical data into a research database, but incorporates feedback to end-users. The data collection is multi-centre and longitudinal, and includes retrospective and prospective data collection, both clinician-reported and client-reported.

### Website

The website for the registry can be found here: https://www.uts.edu.au/treat-registry.

### Ethics

The primary ethics committee that has reviewed and approved the TrEAT Registry is the Hunter New England Local Health District (HNELHD) Human Research Ethics Committee (HREC) (2024/ETH02330). The University of Technology Sydney (UTS) HREC has ratified the ethics approval (ETH25-11008). Ethical approval has been previously provided by other HRECs depending on where the Registry Lead (DM) was employed, including the Macquarie University HREC and the Western Sydney University HREC. To support stability of ethics oversight, and the inclusion of public health sites without requiring multiple ethics applications, the approval for the TrEAT Registry now exists under a National Mutual Acceptance scheme [29]. Under this scheme, private and public clinical sites are approved in the one main ethics application with HNELHD HREC, with each public clinical site in addition requiring only approval from their site-based research governance office through a Site Specific Application (and not separate hospital-based ethics applications).

### Registration

The TrEAT Registry is registered on the Australian Register of Clinical Registries, which lists clinical registries across health and medicine domains in Australia (and often joint Australian and New Zealand activities) and is monitored by the ACSQHC (registration #ACSQHC-ARCR-279). It is tagged as being a registry that addresses the priority domain of “mental health”. The full list of registered clinical registries is accessible here: https://www.safetyandquality.gov.au/our-work/indicators-measurement-and-reporting/national-guidance-clinical-quality-registries/australian-register-clinical-registries.

### End-user involvement in governance

The TrEAT Registry governance structure aligns with recommendations in the National CQR Framework. The legal Governing Body is UTS. The Registry Lead and data custodian (DM) is located at UTS and supported by a Scientific Advisory Committee (co-authors of this paper), which comprises clinicians, people with lived experience, carers, and researchers with diverse backgrounds from across Australia and New Zealand. Quarterly meetings are held with this committee where decisions are made to support the effective functioning of the registry. For current committee members, the reader can refer to the TrEAT Registry website listed above. On a day-to-day basis, central operations are managed by a small operations team located at UTS, comprising the Registry Lead (DM) and a Project Officer (JT), and from time-to-time research students and postdoctoral researchers who have their work embedded in the registry.

### Coverage

The TrEAT Registry is inclusive of outpatient, day patient, residential, and inpatient levels of care in the public and private sector, for both adult and paediatric clients . For up-to-date coverage, including a list of current clinics in the registry, readers may consult the listing for the TrEAT Registry under the Australian Register of Clinical Registries, or see the TrEAT Registry website – links to both are above.

### Consent

Rare for a CQR, the TrEAT Registry seeks explicit consent from all clients receiving treatment at the partner clinics in the registry, or their parent or guardian if < 16 years, following assent from the young person. This is achieved using an online survey platform, which is linked to the participants’ outcome data. Clients (or parents/guardians) consent to their de-identified data being pooled with the data of other clients into a research databank. Where consent is declined, most partner clinics continue to request the client to complete clinical outcome measures and the data are retained within that clinic only to support the treatment of those individuals and not used for research purposes. Clients are also given options to withdraw consent, and to join a mailing list to hear about research opportunities. For rate of consent, see descriptive results below.

### Measures

*Measures in the registry are regularly reviewed*. All sites report on a MDS, providing a consistent set of indicators across services. As a locally-responsive registry, additional time-points or measures are included for specific clinics when requested, or to support specific research projects for set periods of time. At the time of writing, the following MDS is collected at each clinic at treatment commencement and discharge:

*Clinician-reported* Clinician-reported data include diagnoses, type of treatment (level of care, model of care), duration of treatment, frequency of contacts, multidisciplinary team involvement, and medications.

*Self-reported* For clients *≥* 13 years old, data are self-reported and include demographic information, treatment history, and several standardised psychometric questionnaires. The Eating Disorder Examination Questionnaire (EDE-Q; [15]) is a 28-item measure of eating disorder symptoms, widely used globally with Australian clinical cut-offs and community norms for benchmarking [26, 27]. Internal consistency (McDonald’s omega) for the global EDE-Q score for the sample in this protocol is 0.95. The Clinical Impairment Assessment (CIA; [8]) is a 16-item measure of functional impairment associated with eating disorder symptoms. There are established clinical cut-offs and community norms for benchmarking [33]. The internal consistency of the CIA in the current sample is 0.95. The Depression Anxiety and Stress Scale (DASS-21; [23]) is a 21-item measure of symptoms of distress with subscales for depression, anxiety and stress. Clinical cut-offs are provided as a guide by the authors and Australian community norms support benchmarking [18]. The internal consistency of the three scales in the TrEAT Registry has been reported as 0.95, 0.83, and 0.85 for depression, anxiety and stress, respectively.

*Parent-reported.* For clients < 13 years old, data are parent-reported and include demographic information, treatment history, child self-harm, and several standardised measures. These standardised measures include the Children’s Eating Disorder Examination Questionnaire (short version; [21]) as an indicator of eating disorder symptoms; the Strengths and Difficulties Questionnaire (SDQ; [16]) as an indicator of comorbid psychological and behavioural problems; and the Autism Spectrum Quotient Child Version (AQ10; [4]) as an indicator of autistic traits.

### Procedures

*Data Collection* New clients at clinics in the registry are provided an online survey link by staff as part of their usual intake procedures. The link takes clients to a participant information and consent form for the research databank and the pre-treatment survey. Clients are requested to complete the survey measures regardless of whether or not they consent for their data to be pooled in the registry databank. Depending on the agreed time-points, clients are then sent online surveys during treatment (to monitor progress), at the end of treatment, and following the end of treatment. The median time to complete pre-treatment surveys (including reviewing the information and consent form) is 27 min, whereas the median time to complete follow-up surveys is 15 min. Treating clinicians are asked to enter clinician-reported data for each client at each data collection wave on a spreadsheet to be entered into the registry databank.

**Fig. 1:**
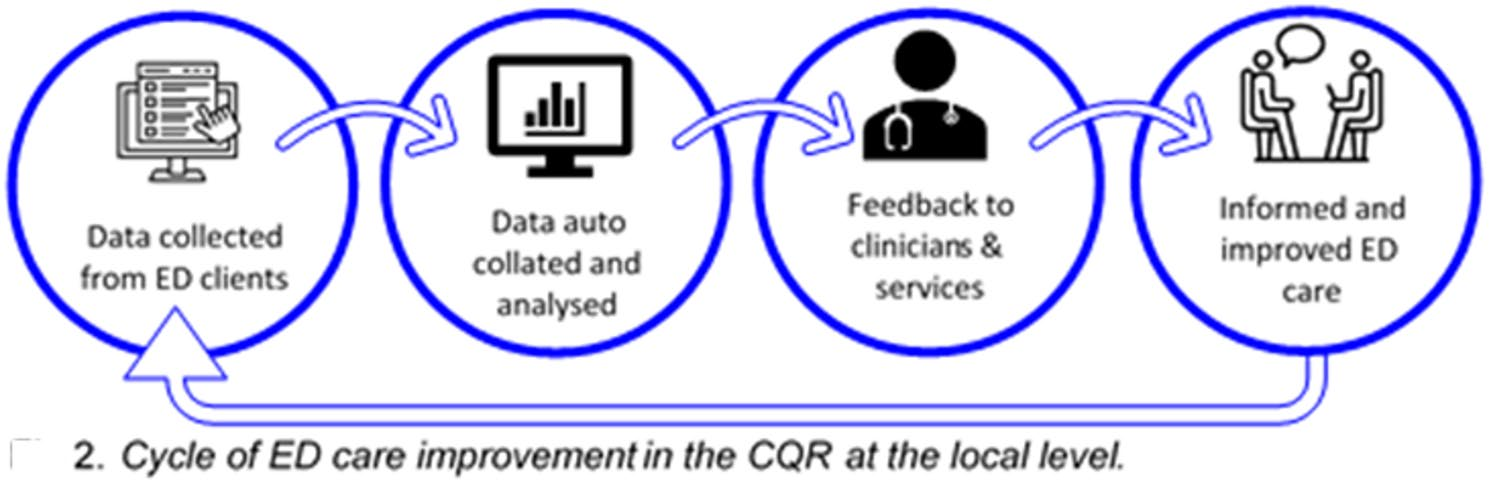
A diagram depicting clinic feedback mechanisms within the TrEAT Registry

*Feedback Mechanisms.* A core purpose of the TrEAT Registry is to support quality improvement by reporting back to registry end-users to aid data-informed decision-making (see Fig. 1).

The current reporting processes include:

- *Benchmark Reporting to Clinicians.* After a client (or parent/guardian) completes a survey, scoring of the measures occurs in real-time automatically. The raw data and scores are then immediately sent via email to the client’s clinic. Alongside scores on the measures, clinics receive information on established clinical cut-offs and community norms for each measure, to help them to detect outliers and monitor clinical progress against expected standards, and to determine if other specialist services may be indicated for referral.
- *Benchmark Reporting to Clinics.* Clinics can request access to their raw data to support quality assurance and improvement activities. From time-to-time the TrEAT Registry has provided benchmarking reports to services that summarises client outcome data and benchmarks this against the mean scores for similar clinics in the registry. With increased resourcing, this is intended to become a core process within the registry, either supported by reports sent from the central research team to services, or through an automated digitised report-builder that clinicians can access directly.
- *Registry-Wide Reporting.* Where resources have allowed, an annual report of outcomes across the entire registry has been shared with partner clinics. The intention for the future is to make these reports public-facing, published on the TrEAT Registry website.

### Data governance

The TrEAT Registry has a data governance framework in place to protect client privacy that is aligned with the National CQR Framework [1] and international privacy standards and has been approved by the relevant ethics committees and research governance offices. Data that are held in the registry databank are de-identified. The only identifying information that is collected by the central registry team is the participants’ contact email address and date of birth, and these data are removed and stored in a separate spreadsheet away from the clinical information in the databank. The data are stored on a secure platform under multi-factor authentication and password protection, accessible only to the central registry operating team. Data sharing for secondary analysis is facilitated through a process where researchers submit a proposal to the Registry Lead for the use of the data, and if deemed appropriate, with evidence of ethical clearance, this is reviewed by the relevant clinics who can opt in or out of sharing the data from their clinic. Embedded registry research is also encouraged, such as registry-based clinical trials, and undergoes the same proposal process and a separate ethics application.

### Partners

The TrEAT Registry is supported in-kind by its partner, the Australia & New Zealand Academy for Eating Disorders (ANZAED). ANZAED is the peak body representing eating disorder professionals across Australia and New Zealand and manages the Eating Disorder Credential in Australia (www.connected.anzaed.org.au). The ANZAED Eating Disorder Credential is a government-funded scheme that recognises and creates a directory of clinicians who meet minimum training and supervision standards for delivering safe and effective eating disorder treatment. The registry is also supported in-kind and financially (in the case of public and large private facilities) by the partner clinics, with an up-to-date list available on the website.

## Results

Data were pulled from the registry databank for clients aged 13 years or older over the period September 2021 to June 2025. This period of time was chosen as it reflected relative stability in the number of clinics and variables in the registry. Over this time, 754 clients were provided information about the registry and clicked through to the online survey link. Of these clients, *N* = 664 (88.1%) consented for their data to be added to the research databank and 662 (99.7%) also went on to respond to the pre-treatment survey questions. Clients < 13 years were excluded from this descriptive analysis to preserve confidentiality given there were only *n* = 10 who completed the pre-treatment survey questionnaires in this age group over the data capture period.

### Clinic level descriptive characteristics

There were 10 clinics in the registry over this period of time for which data were captured. This included eight outpatient clinics (contributing 76.5% of the data), one day program, and one residential program. All outpatient clinics were privately operated, whereas the day program is run as a fee-free service by a non-government organisation, and the residential program has a mixture of public and private beds. Regarding geographic spread, while there is just one of the 10 clinics in New Zealand, this clinic has four sites and contributed 27.8% of the data. Within Australia, two of the clinics are in regional areas of Queensland, five in urban New South Wales, one in urban Western Australia, and one is delivered online. Expressions of interest to join the registry are continually received and processed. Since this data capture period, four additional sites have commenced data collection including two public residential clinics in urban New South Wales and Victoria, and one private inpatient and one private outpatient clinic in urban New South Wales.

### Client demographic and clinical characteristics

Table 1 presents descriptive statistics on the demographic and clinical characteristics of the sample.

**Table 1:** Demographic and clinical characteristics of clients (*≥* 13 years) in the TrEAT registry research databank taken from the pre-treatment (baseline) survey, statistics are mean (standard deviation) for continuous variables and n (%) for categorical variables

Age and incidence of eating disorder	
Current age (*n* = 618)	26.0 (10.5), 13–77 years
Age onset symptoms (*n* = 590)	15.5 (6.1)
Age diagnosed with ED (*n* = 595)	22.2 (9.9)
Years since ED diagnosis (*n* = 565)	3.5 (6.3)
**Location and demographic information**	
Urbanicity (Australia) (*n* = 429)	
Major City	371 (86.5)
Inner Regional	45 (10.5)
Outer Regional	13 (3.0)
Urbanicity (New Zealand) (*n* = 167)	
Major Urban Area	146 (87.4)
Medium to Large Urban Area	16 (9.6)
Small Urban Area/Rural Settlement	5 (3.0)
Gender (*n* = 622)	
Girl/woman	571 (91.8)
Boy/man	43 (6.9)
Other^†^	8 (1.3)
Region of birth (*n* = 621)	
Australia	374 (60.2)
New Zealand	160 (25.8)
Europe	45 (7.2)
Asia	11 (1.8)
Americas	10 (1.6)
Africa	5 (0.8)
Other	16 (2.6)
Indigenous identity	
Aboriginal (*n* = 621)	6 (1.0)
Torres Strait Islander (*n* = 621)	0 (0.0)
Māori or Pacific Islander (*n* = 620)	14 (2.4)
Relationship Status (*n* = 621)	
Never married	487 (78.5)
Married	62 (10.0)
Separated/divorced	23 (3.7)
Other^†^	2 (0.3)
Occupation (*n* = 621)	
Studying	271 (43.6)
Full time work	164 (26.4)
Part time work	83 (13.4)
Home duties	19 (3.1)
Unemployed	36 (5.8)
Not working due to disability	26 (4.2)
Other	22 (3.6)
Qualification (*n* = 621)	
Bachelor degree or higher	249 (40.1)
Still at school	135 (21.7)
Certificate/diploma	98 (15.8)
Still studying at university, college etc.	95 (15.3)
Secondary school (left > 15 years)	26 (4.2)
Secondary school (left < 16 years)	10 (1.6)
Household income per annum (*n* = 577)	
$0 - $50,000	100 (17.3)
$50,001 - $100,000	104 (18.1)
> $100,000	204 (35.4)
Unsure	169 (29.3)

Table 2 displays the mean scores and standard deviations on the core patient-reported outcome measures used in the registry. Established data on clinical norms and cut-offs are provided for contextual purposes.

**Table 2:** Means and standard deviations of the core patient-reported outcome measures at baseline in the registry sample, with descriptive comparison to published community norms and clinical cut-offs

	Mean (standard deviation)		Clinical cut-off	Reliability in the registry (ω)
	Registry sample	Community norms		
EDE-Q (*n* = 662)				
Global	3.80 (1.44)	1.52 (1.25)^1^	2.3^2^	0.95
Restraint	3.24 (1.85)	1.30 (1.40) ^1^	-	0.88
Eating concern	3.31 (1.44)	0.76 (1.06) ^1^	-	0.74
Weight concern	4.16 (1.63)	1.79 (1.51) ^1^	-	0.85
Shape concern	4.48 (1.58)	2.23 (1.65) ^1^	-	0.92
CIA total score (*n* = 642)	30.92 (12.03)	6.4 (7.5)^3^	16^4^	0.95
DASS-21 (*n* = 556)				
Depression	10.29 (5.83)	2.83 (3.87)^5^	11^6^	0.95
Anxiety	7.00 (4.66)	1.88 (2.95) ^5^	8^6^	0.83
Stress	10.67 (4.79)	4.73 (4.20) ^5^	13^6^	0.85

### Previous treatment

Regarding previous health service use related to eating disorder treatment, across the sample, 484 (83.9%) reported having attended at least one appointment with a general practitioner, 415 (72.7%) reported having attended at least one therapy appointment, 394 (66.7%) reported having attended at least one dietitian appointment, and 242 (36.9%) clients reported having at least one hospital admission for their eating disorder. Estimates for exact number of previous eating disorder treatment episodes/appointments ranged considerably, which may reflect the difficulty of lifetime recall for such precise information. For instance, number of previous hospitalisations ranged from 0 to 150 (median = 0), number of previous therapy and dietetic sessions both ranged from 0 to 2000 (median = 6 and 2, respectively), and number of previous appointments with a general practitioner ranged from 0 to 5000 (median = 4).

## Discussion

The TrEAT Registry is an important step forward in eating disorder health data surveillance in Australia and New Zealand. The planned expansion of this registry to achieve greater bi-national coverage across public and private specialist clinics will support a “learning health system”, whereby collection, collation, benchmarking and feedback of data to all end-users will ultimately lead to responsive and improved eating disorder care. Registry governance is overseen by a committee that intentionally includes clinicians, people with lived experience, policy workers and researchers, with diversity across gender, location, private/public sector, and discipline. This is critical to ensuring that the needs of all end-users are considered in registry decision-making. As a critical piece of health data infrastructure, this registry provides a valuable tool for researchers and funders, to reduce the costs of clinical trials and data linkage projects, and to accelerate translation of research to practice.

Descriptive data from the baseline survey over the past few years for clients aged *≥* 13 years demonstrates that the registry databank is broadly representative of eating disorder treatment-seeking samples. This includes a demographic distribution characterised as young (M ± 1 SD = 15.5–36.5 years), mostly girls/women (9 in 10), and residing in urban regions (> 85%). While this is a similar distribution to clinical samples in the published literature (e.g., [31]), epidemiological research using population-based samples has provided evidence that the young white urban female stereotype of who experiences disordered eating is not so pronounced in the community [9, 25]. What this demonstrates is the existence of significant barriers to accessing eating disorder care among those who do not fit the notion of who typically experiences an eating disorder. By regularly collating reports on who is accessing care for an eating disorder in specialist clinics, the TrEAT Registry will be a valuable tool to monitor the treatment gap and guide interventions to improve access to care for all.

One gap that can be investigated through the registry is access to culturally safe eating disorders care among Indigenous people, including Aboriginal and/or Torres Strait Islander peoples and Māori. Despite knowledge of healthcare inequities for Indigenous populations, over 70% of clinical registries in Australia do not collect information on Indigenous identification [35], which is a significant barrier to supporting community-led actions to address equity concerns. In a current extensive review of the TrEAT Registry, we are considering how the registry can best align with Indigenous data sovereignty principles, in regard to culturally sensitive and effective approaches to data collection, analysis and reporting. Ultimately it is hoped that this will support community-centred approaches to eating disorder healthcare access and outcomes for Indigenous peoples.

The TrEAT Registry is unique in its coverage of specialist private practices among its included services. There are two main reasons why we have been successful in this. Firstly, the registry was conceived of, and designed by, a group of clinical leaders who are also private practice owners. “Grass roots” clinical leadership is a known factor in the take up and sustainability of registries [2]. Secondly, the TrEAT Registry is supported by ANZAED, the peak bi-national professional organisation that brings together and supports eating disorder clinicians, across public and private sectors. Their support of the registry is valuable for reaching and engaging additional clinics during the next five years of strategic growth. As a bi-national body, ANZAED’s support is also instrumental in achieving coverage across Australia and New Zealand. Another important distinguishing feature of the TrEAT Registry is that consent is sought from clients and carers, which is not typically required for quality assurance work. This was deemed important from the outset in the design of the registry in respecting the rights of clients to know how their information is being collected and to be able to opt out of those uses. Not only is this intrinsically a more ethical practice, but it facilitates trust in the registry among the lived experience community and ultimately supports a high level of consent (88%).

### Limitations

Limitations of the TrEAT Registry include those that are experienced broadly across registries. Namely, the registry has incomplete coverage of the sector. This limits the reliability and utility of benchmarking exercises. On the other hand, the demographic distribution and scores on clinical measures suggests that the current registry sample is representative of people seeking treatment for an eating disorder. Furthermore, while baseline data collection is consistently high, follow-up data collection is inconsistent. For example, while follow-up and discharge data are consistently collected at day program and residential clinics in the registry, rates of completing patient-reported outcome measures in the private outpatient clinics is closer to 25%. This limits meaningful analysis of treatment outcomes, including examination of predictors of best outcomes. While some of this can be overcome with collecting data from clinicians as well as from clients, and through data linkage (for example to other health service use outcomes), it is a priority to improve follow-up data completeness. While the descriptive data for most of the variables were in keeping with expectations for a clinical eating disorder sample, the large ranges for the questions regarding previous healthcare appointments were unexpected, and may suggest that lifetime recall of such information is unreliable and the way these questions are asked should be reviewed. We identified few children receiving treatment for an eating disorder who are younger than 13 years in the registry. The true age distribution for eating disorders remains elusive given the age ranges of many population-based surveys which rarely extend below 13 years. It is therefore possible that their low numbers in the registry represents one of a number of possibilities including low base rates of eating disorders in young children in the general population, under-detection and treatment of eating disorders in this age group, under-representation of younger children with eating disorders in the private sector, or low rates of consent from parents attending clinics in the registry to add their child’s data to a registry databank. Work is currently being done to develop better screening measures to detect problems with eating in younger age groups [20]. We also anticipate that as the number of children in the registry databank grows over time that the database will be a valuable mechanism to better understand the phenomenology and best treatment approaches for younger children. Other technical limitations include the reliance on manual processes for creating clinic benchmark reports, and the lack of access for clinicians and clients to visualisations of their outcome data. Finally, a contextual challenge that affects the sustainability of the registry is availability of funding for its continued operations.

### Future directions

Recent grant funding from the Australian government has been secured for the next five years to digitally enhance and expand the coverage of the TrEAT Registry. A major goal for this period includes working with all end-users to design and build a new digital architecture that will address identified limitations, including clinic access to summarised data in visual format, automated report building, and a notification system to improve follow-up data collection. Over this time, we will also focus on recruiting additional public and private eating disorder clinic sites to improve coverage of the sector. Finally, the registry itself will be used as a tool to support research. It is hoped that by achieving these outcomes, a case can be made for continued government support for the registry, aligned with the National CQR Strategy [7].

Beyond these shorter-term goals, we intend to create a successful model for eating disorder registries that can be used to support the development of registries elsewhere in the world, aspiring to an internationally synchronised database of real-world eating disorder treatment registries across the globe.

## Conclusions

The TrEAT Registry offers a valuable resource for monitoring the safety, equity and effectiveness of eating disorders treatment in Australia and New Zealand.

## Data Availability

Available on request and subject to approvals from the first author.

## References

[1] ACSQHC. Prioritised list of clinical domains for clinical quality registry development: final report. Sydney: ACSQHC; 2016.

[2] Ahern S, Gabbe BJ, Green S, Hodgson CL, Wood EM, Oam Z, J. R., Zazryn T. Realising the potential: leveraging clinical quality registries for real world clinical research. Med J Australia. 2022;216(6):273–7. 10.5694/mja2.51443. 35267192 10.5694/mja2.51443

[3] Allison C, Auyeung B, Baron-Cohen S. Toward brief red flags for autism screening: the short autism spectrum quotient and the short quantitative checklist for autism in toddlers in 1,000 cases and 3,000 controls [corrected]. J Am Acad Child Adolesc Psychiatry. 2012;51(2):202–e2127. 10.1016/j.jaac.2011.11.003. 22265366 10.1016/j.jaac.2011.11.003

[4] Australian Department of Health. National Clinical Quality Registry and Virtual Registry Strategy*.* Publications No:12732. 2020.

[5] Australian Commission on Safety and Quality in Health Care. Australian Framework for National Clinical Quality Registries. Sydney. 2024.

[6] Australian Commission on Safety and Quality in Health Care. Framework for Australian Clinical Quality Registries – Consultation Version. Sydney. 2022.

[7] Australian Institute of Health and Welfare. Medicare mental health services*.* AIHW. 2025. https://www.aihw.gov.au/mental-health/topic-areas/medicare-subsidised-services

[8] Bohn K, Doll HA, Cooper Z, O’Connor M, Palmer RL, Fairburn CG. The measurement of impairment due to eating disorder psychopathology. Behav Res Ther. 2008;46(10):1105–10. 18710699 10.1016/j.brat.2008.06.012 PMC2764385

[9] Burt A, Mannan H, Touyz S, Hay P. Prevalence of DSM-5 diagnostic threshold eating disorders and features amongst aboriginal and Torres Strait Islander peoples (First Australians). BMC Psychiatry. 2020;20(1):449. 10.1186/s12888-020-02852-1. 32917167 10.1186/s12888-020-02852-1 PMC7488483

[10] Center for Psychiatric Research. National Quality Register for Eating Disorder Treatment. 2025.

[11] Commonwealth of Australia. More support for Australians with eating disorders. Health. 2022. https://www.health.gov.au/ministers/the-hon-david-coleman-mp/media/more-support-for-australians-with-eating-disorders

[12] Day S, Hay P, Basten C, Byrne S, Dearden A, Goldstein M, Hannigan A, Heruc G, Houlihan C, Roberts M, Tannous WK, Thornton C, Valentine N, Mitchison D. Posttraumatic stress disorder (PTSD) and complex PTSD in eating disorder treatment-seekers: prevalence and associations with symptom severity. J Trauma Stress. 2024;37(4):672–84. 10.1002/jts.23047. 38637955 10.1002/jts.23047

[13] Day S, Mitchison D, Tannous WK, Conti J, Gill K, Le L, Mannan H, Mihalopoulos C, Ramjan L, Rankin R, Hay P. Longitudinal effects of residential treatment for eating disorders: symptom trajectories and predictors of functional outcomes. Int J Eat Disord. 2025;58(7):1367–80. 10.1002/eat.24448. 40271727 10.1002/eat.24448 PMC12232342

[14] Deloitte Access Economics. Paying the Price, Second Edition: The economic and social impact of eating disorders in Australia. February 2024. 2024.

[15] Fairburn CG, Beglin SJ. Eating Disorder Examination Questionnaire (6.0). In Fairburn CG. Cognitive Behavior Therapy and Eating Disorders. New York: Guilford Press. 2008.

[16] Goodman R. The strengths and difficulties questionnaire: a research note. J Child Psychol Psychiatry. 1997;38(5):581–6. 9255702 10.1111/j.1469-7610.1997.tb01545.x

[17] Hamilton A, Mitchison D, Basten C, Byrne S, Goldstein M, Hay P, Heruc G, Thornton C, Touyz S. Understanding treatment delay: perceived barriers preventing treatment-seeking for eating disorders. Aust N Z J Psychiatry. 2022;56(3):248–59. 10.1177/00048674211020102. 34250844 10.1177/00048674211020102

[18] Henry JD, Crawford JR. The short-form version of the Depression Anxiety Stress Scales (DASS-21): construct validity and normative data in a large non-clinical sample. Br J Clin Psychol. 2005;44(Pt 2):227–39. 10.1348/014466505X29657. 16004657 10.1348/014466505X29657

[19] Heruc G, Hurst K, Trobe S, et al. Development and implementation of a credentialing system for clinicians providing eating disorder care. J Eat Disord. 2025;13(Suppl 1):144. 10.1186/s40337-025-01310-1. 40676641 10.1186/s40337-025-01310-1 PMC12272996

[20] Jabs M, Pennesi JL, Baillie S, Hay P, Mitchison D, Norton L, Prnjak K, Wade TD, Hart L. Validated eating disorder screening tools for paediatric populations: A systematic review. Psychiatry Res. 2025;351:116631. 10.1016/j.psychres.2025.116631. 40706277 10.1016/j.psychres.2025.116631

[21] Kliem S, Schmidt R, Vogel M, Hiemisch A, Kiess W, Hilbert A. An 8-item short form of the eating disorder Examination-Questionnaire adapted for children (ChEDE-Q8). Int J Eat Disord. 2017;50(6):679–86. 10.1002/eat.22658. 28122128 10.1002/eat.22658

[22] Linardon, et al. The efficacy of cognitive-behavioral therapy for eating disorders: A systematic review and meta-analysis. J Consulting Clin Psychol. 2017;85(11):1080–94. 10.1037/ccp0000245 29083223

[23] Lovibond S, H., Lovibond P, F. Manual for the depression anxiety stress scales. 2nd ed. Sydney: Psychology Foundation; 1995.

[24] McLean S, Fuller-Tyszkiewicz M, et al. Evaluation of the eating disorders medicare benefit schedule items 2024. Prepared for the Australian Government Department of Health and Aged Care; 2024.

[25] Mitchison D, Hay P, Slewa-Younan S, Mond J. The changing demographic profile of eating disorder behaviors in the community. BMC Public Health. 2014;14:943. 10.1186/1471-2458-14-943. 25213544 10.1186/1471-2458-14-943 PMC4246495

[26] Mond JM, Hay PJ, Rodgers B, Owen C, Beumont PJ. Validity of the eating disorder examination questionnaire (EDE-Q) in screening for eating disorders in community samples. Behav Res Ther. 2004;42(5):551–67. 10.1016/S0005-7967(03)00161-X. 15033501 10.1016/S0005-7967(03)00161-X

[27] Mond JM, Hay PJ, Rodgers B, Owen C. Eating Disorder Examination Questionnaire (EDE-Q): norms for young adult women. Behav Res Ther. 2006;44(1):53–62. 10.1016/j.brat.2004.12.003. 16301014 10.1016/j.brat.2004.12.003

[28] Murray, et al. Treatment outcomes for anorexia nervosa: a systematic review and meta-analysis of randomized controlled trials. Psychol Med. 2019;49(4):535–44. 30101734 10.1017/S0033291718002088

[29] National Health & Medical Research Council. *National Certification Scheme for the ethics review of multi-centre research.* NHMRC. 2025. https://www.nhmrc.gov.au/research-policy/ethics/national-certification-scheme-ethics-review-multi-centre-research

[30] New Zealand Psychologists Board. Annual report 2023–2024. 2024. https://psychologistsboard.org.nz/wp-content/uploads/2025/02/New-Zealand-Psychologists-Board-Annual-Report-2024.pdf

[31] Pellizzer ML, Waller G, Wade TD. A pragmatic effectiveness study of 10-session cognitive behavioural therapy (CBT-T) for eating disorders: targeting barriers to treatment provision. Eur Eat Disorders Rev. 2019;27(5):557–70. 10.1002/erv.2684. 10.1002/erv.2684 31134731

[32] Platt JE, Raj M, Wienroth M. An analysis of the learning health system in its first decade in practice: scoping review. J Med Internet Res. 2020;22(3):e17026. 10.2196/17026. 32191214 10.2196/17026 PMC7118548

[33] Reas DL, Stedal K, Lindvall Dahlgren C, Rø Ø. Impairment due to eating disorder pathology: Identifying the cut-off score on the Clinical Impairment Assessment in a clinical and community sample. Int J Eat Disord. 2016;49(6):635–8. 10.1002/eat.22517. 26968998 10.1002/eat.22517

[34] Russell P, Potter E, Heruc G, Anderson J. Urgent call to action: mobilising physicians and the medical workforce to address treatment of medical instability from eating disorders. Intern Med J. 2025;55(9):1423–6. 10.1111/imj.70189. 40888189 10.1111/imj.70189

[35] Ryder C, Hossain S, Howard L, Severin J, Ivers R. Indigenous governance, ethics and data collection in Australian clinical registries. Med J Aust. 2024;221:156–61. 10.5694/mja2.52383. 38984375 10.5694/mja2.52383

[36] Santomauro DF, Melen S, Mitchison D, Vos T, Whiteford H, Ferrari AJ. The hidden burden of eating disorders: an extension of estimates from the Global Burden of Disease Study 2019. Lancet Psychiatry. 2021;8(4):320–8. 10.1016/S2215-0366(21)00040-7. 33675688 10.1016/S2215-0366(21)00040-7 PMC7973414

[37] School of Public Health and Preventive Medicine, Monash University. Public health and preventive medicine: A guide for registry-based trials. Version 1.0, December 10, 2024. Melbourne, Australia: Monash University. 2024. https://www.health.gov.au/resources/publications . Accessed 28/6/25.

[38] Te Pou o te Whakaaro Nui. 2024 Health New Zealand Te Whatu Ora adult workforce estimates. 2024. https://www.tepou.co.nz/resources/2024-health-new-zealand-te-whatu-ora-adult-workforce-estimates

